# Modeling of Virion Collisions in Cervicovaginal Mucus Reveals Limits on Agglutination as the Protective Mechanism of Secretory Immunoglobulin A

**DOI:** 10.1371/journal.pone.0131351

**Published:** 2015-07-01

**Authors:** Alex Chen, Scott A. McKinley, Feng Shi, Simi Wang, Peter J. Mucha, Dimple Harit, M. Gregory Forest, Samuel K. Lai

**Affiliations:** 1 Departments of Mathematics and Applied Physical Science, University of North Carolina–Chapel Hill, Chapel Hill, NC, 27599–3250, United States of America; 2 Mathematics Department, University of Florida, 358 Little Hall, PO Box 118105, Gainesville, FL, 32611–8105, United States of America; 3 Division of Molecular Pharmaceutics, Eshelman School of Pharmacy, University of North Carolina–Chapel Hill, 120 Mason Farm Road, Chapel Hill, NC, 27599–7362, United States of America; 4 UNC/NCSU Joint Department of Biomedical Engineering, University of North Carolina–Chapel Hill, 120 Mason Farm Road, Chapel Hill, NC, 27599, United States of America; University of California Irvine, UNITED STATES

## Abstract

Secretory immunoglobulin A (sIgA), a dimeric antibody found in high quantities in the gastrointestinal mucosa, is broadly associated with mucosal immune protection. A distinguishing feature of sIgA is its ability to crosslink pathogens, thereby creating pathogen/sIgA aggregates that are too large to traverse the dense matrix of mucin fibers in mucus layers overlying epithelial cells and consequently reducing infectivity. Here, we use modeling to investigate this mechanism of “immune exclusion” based on sIgA-mediated agglutination, in particular the potential use of sIgA to agglutinate HIV in cervicovaginal mucus (CVM) and prevent HIV transmission. Utilizing reported data on HIV diffusion in CVM and semen, we simulate HIV collision kinetics in physiologically-thick mucus layers–a necessary first step for sIgA-induced aggregation. We find that even at the median HIV load in semen of acutely infected individuals possessing high viral titers, over 99% of HIV virions will penetrate CVM and reach the vaginal epithelium without colliding with another virion. These findings imply that agglutination is unlikely to be the dominant mechanism of sIgA-mediated protection against HIV or other sexually transmitted pathogens. Rather, we surmise that agglutination is most effective against pathogens either present at exceedingly high concentrations or that possess motility mechanisms other than Brownian diffusion that significantly enhance encounter rates.

## Introduction

Plasma cells secrete polymeric IgA (pIgA), predominantly as dimers in which two IgA monomers are covalently linked by the joining (J) chain [1]. The polymeric immunoglobulin receptors on epithelial cells subsequently bind pIgA present in the basolateral membrane, undergo transcytosis, and release secretory IgA (sIgA, comprising pIgA and the secretory component) into mucus overlaying the apical membrane. Since sIgA is predominantly found in mucus secretions while almost non-existent in serum [2], sIgA is generally considered to be the major antibody class associated with mucosal protection. Indeed, many studies have correlated increases in viral-specific sIgA levels at the mucosal surface with either reduced virus shedding, or protection against infection and disease [3]. sIgA is thought to be well suited for mucosal protection in part because the secretory component can help reduce degradation by proteases in mucus. sIgA also possesses little to no complement activating or phagocytic uptake simulating ability [4, 5], which limits inflammation-induced damage to the epithelium. Last but not least, in addition to protection by binding to neutralizing epitopes, the polymeric nature of sIgA also facilitates protection by “immune exclusion”–the agglutination of microorganisms by polymeric immunoglobulins (antibodies) into clusters too large to diffuse through mucus [6]. The relevance of sIgA-mediated immune exclusion in the female reproductive tract is vividly illustrated by agglutination of otherwise vigorously motile sperm, which make little to no forward progress after agglutination and cannot ‘swim’ across mucus [7].

Given the association between IgA and mucosal protection, recent efforts to develop effective strategies to block vaginal HIV transmission have included vaccines aimed to induce sIgA response in the female reproductive tract [8, 9], IgA immunoprophylaxis using adeno-associated viral vectors [10], and hematopoietic stem/progenitor cells pre-transduced with an appropriate IgA gene [11]. Since CVM secretions contain far more IgG than sIgA [12], a major premise in these ongoing efforts is that sIgA may provide improved protection and better reinforce the first line of defense (i.e., mucus) against HIV infections than IgG. In theory, Fab domains on IgG and sIgA with similar affinity to HIV-Env should bind and neutralize HIV virions with comparable potencies. We therefore are led to question whether sIgA-induced agglutination of pathogens may contribute additional protection against HIV and other sexually transmitted viral infections in the female reproductive tract. Due to the technical challenges associated with visualizing the agglutination of a homogeneous population of gp120+ fluorescently tagged HIV virions in mucus secretions in real-time, we employ rigorous modeling and simulations over the physiologically relevant parameter space to address this question. This approach provides quantitative insights into this dynamic process, and enables us to explore the precise conditions (virion and antibody concentration, diffusive properties, mucus layer thickness and drainage times, etc.) where agglutination may afford significant protection.

## Materials and Methods

**Fig 1:**
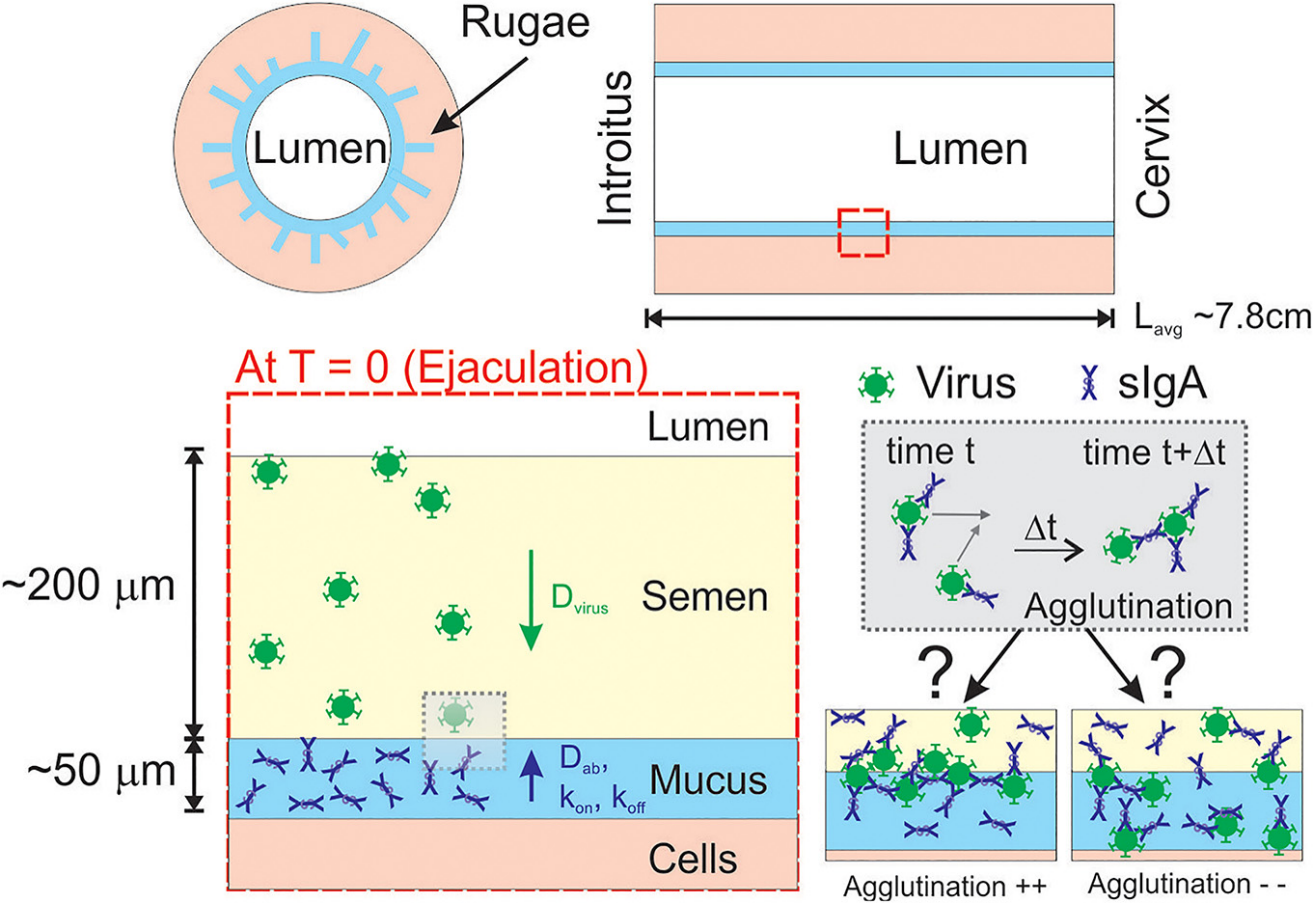
Schematic of our model. The schematic depicts diffusion of HIV from seminal secretions across the cervicovaginal mucus (CVM) layer containing HIV-binding sIgA en route to the underlying vaginal epithelium. For sIgA-induced agglutination to occur, virions must collide with each other. Virions and antibodies are not drawn to scale.

We model a virus population by a vector $\vec{V}\left(z,t\right)$, where the component *V*_*n*_(*z*, *t*) represents the concentration of agglutinated complexes of *n* virions at a spatial location *z* and time *t*. HIV virions in semen can be broadly categorized as cell-free (individual viruses) and cell-associated (e.g. associated with leukocytes); to establish an upper limit on agglutination of individual virions, we undertook the extreme assumption that all HIV viruses in semen exist as individual virions. By assuming that HIV do not associate with cells, and based on prior evidence that shows HIV is readily mobile in semen (implying no association to mucins or other matrix components) [13], we also assume that individual HIV virions are uniformly distributed within the semen layer *d* < *z* < *L* initially. We track the population the $\vec{V}\left(z,t\right)=\;\left(V_{1}\left(z,t\right),V_{2}\left(z,t\right),\ldots ,V_{M}\;\left(z,t\right)\right)$ in the semen-CVM system 0 < *z* < *L* over time. For computational purposes, the maximum number of virions in a complex is limited to a value *M*, which may be considered as tracking the concentration of agglutinated *M*-complexes or larger. For most of the simulation results, the concentration of agglutinated virus for complexes larger than *M* is negligible, making this approximation accurate. We incorporate two effects for the interaction of virus in the system. First, the virus population diffuses within the system, with a reflecting boundary condition at the semen-air interface (*z* = *L*) and an absorbing condition at the epithelial layer (*z* = 0) in order to account for the fact that HIV virions that can infect the host must bind to exposed target cells or penetrate into the sub-epithelium. Simultaneously, the complexes of the virus population react with each other, agglutinating by sIgA crosslinking (Fig 1). We ignore effects such as gp120 shedding (T_1/2_, 30 hrs) and thermal degradation from RNA polymerase decay (T_1/2_, 40 hrs) because of the substantial difference in the rate of these processes from the time scale of interest [14], and would only decrease the viral load or agglutination rate. Substitution of the absorbing condition with a reflecting condition at the epithelial cell layer does not markedly increase agglutination rates (data not shown).

By assuming that every encounter results in a successful agglutination, we can utilize the number of encounters to establish an upper bound on the highest possible agglutination rate of virus. The Smoluchowski encounter rate [15] is a formula describing the collision rate of two concentrations of spherical particles diffusing freely in three dimensions. For concentrations *C*_1_, *C*_2_ of particles of hydrodynamic radii *r*_1_, *r*_2_ and diffusion coefficients *d*_1_, *d*_2_, the encounter rate is *k* = 4*π*(*d*_1_ + *d*_2_)(*r*_1_ + *r*_2_)*C*_1_ *C*_2_. These effects can be represented by the Smoluchowski coagulation equation, a reaction-diffusion system:

$$\frac{\partial \vec{V}}{\partial t}=D\frac{\partial^{2}\vec{V}}{\partial z^{2}}-\;\vec{f}\left(\vec{V}\;\left(z,t\right)\right)+\vec{g}\left(\vec{V}\left(z,t\right)\right),$$

where $f_{n}\left(\vec{V}\left(z,t\right)\right)=\sum_{j=1}^{M-n}4\pi \left(D_{j}+D_{n}\right)\left(r_{j}+r_{n}\right)V_{j}V_{n}+1_{\left\{n\leq M/2\right\}}16\pi D_{n}r_{n}V_{n}^{2}$ and

$$g_{n}\left(\vec{V}\left(z,t\right)\right)=\sum_{j=1}^{n-1}4\pi \left(D_{j}+D_{n-j}\right)\left(r_{j}+r_{n-j}\right)V_{j}V_{n-j},$$ (1)

where the diffusion tensor ***D***, is a diagonal tensor with entries *D*_1_,*D*_2_,…,*D*_*M*_ along the diagonal. These entries are calculated depending on the hydrodynamic radius of each complex, described in the following section. An absorbing condition is used at the CVM-epithelial interface $\left(\vec{V}\left(0,t\right)=\vec{0}\right)$ and a reflecting condition is used at the semen-air interface $\left(\frac{\partial \vec{V}}{\partial t}\left(L,t\right)=\vec{0}\right).$

The reaction terms *f*_*n*_ and *g*_*n*_ represent the collision of virions resulting in agglutination. The first term in *f*_*n*_ subtracts the Smoluchowski encounter rate for *j*-complexes and *n*-complexes. Since the largest complex size is *M*, *M* − *n* is the maximum possible value for *j*. The second term reflects the additional depletion of the concentration of *n*-complexes as a result of collisions between *n*-complexes and *n*-complexes. The indicator variable 1_{*n*≤*M* / 2}_ in this term indicates that the term is nonzero only when *n* ≤ *M* / 2, i.e. two *n*-complexes will only agglutinate when the resulting 2*n*-complex is smaller than or equal to the maximum complex size *M*. The increase in *n*-complex concentration is given by the collision of (*n* − *j*)-complexes with *j*-complexes, reflected in the summation for *g*_*n*_. In order to calculate an upper bound for agglutination, we assume that the reverse reaction—that of an *n*-complex uncoupling to become an *j*-complex and (*n* − *j*)-complex—does not occur.

**Fig 2:**
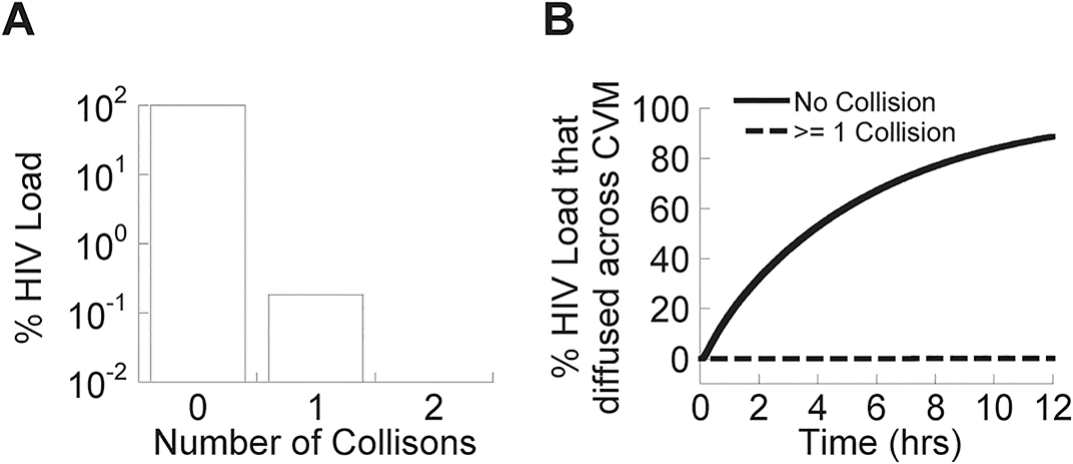
Estimating the number of collisions of HIV virions. The number of collisions between (A) HIV virions in the vagina, and (B) HIV virions that have diffused across CVM and reached the vaginal epithelium, over the first 12 hours post deposition, assuming a viral load of 4.5 log_10_ RNA copies per mL, the mean peak viral load for HIV in semen of acutely infected males most likely to transmit HIV [16].

**Fig 3:**
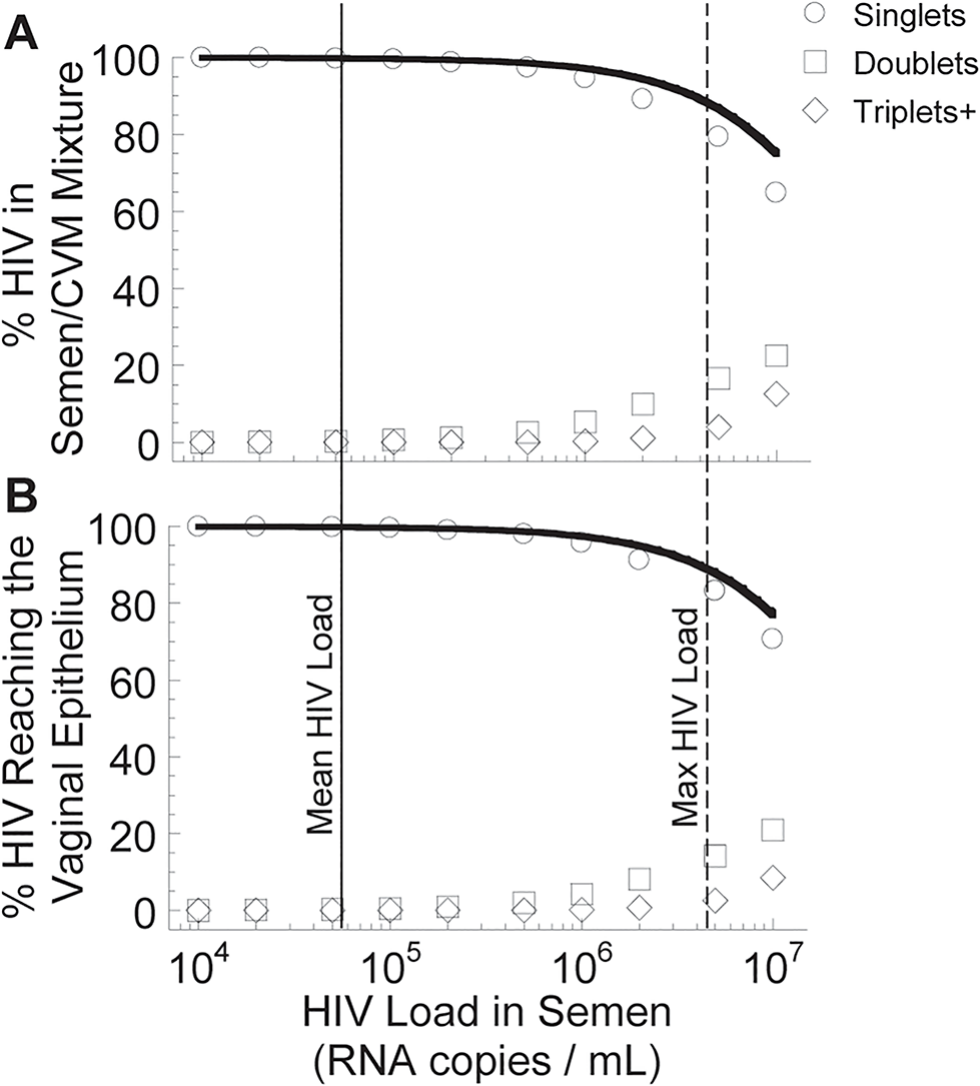
Maximum HIV collision rates for peak HIV concentration. The fraction of HIV virions in ejaculate that have undergone at least one, two, as well as three or more collisions over the first 12 hours post ejaculation as a function of HIV virion density in semen for (A) all HIV virions in the female reproductive tract, and (B) HIV virions that have diffused across CVM and reached the vaginal epithelium. Mean and maximum HIV viral load in semen is obtained from [16]. To enable rapid estimation of the viral load that may result in significant rates of agglutination, we also derived a mathematical equation (dashed curve; first summand of Eq 10) and utilized an exponential approximation (solid curve; Eq 3) that directly estimate the virion load arriving at the epithelial layer that experiences no collisions with other virions (see Appendix and S1 File).

The reaction-diffusion system was simulated with a Forward Time-Central Space scheme in one spatial dimension. Since the Smoluchowski encounter rate equation is valid for spherical particles in three dimensions, the simulation is a one-dimensional projection of the dynamics of the three-dimensional system. The simulations were also verified by an analytic estimate of the virion load arriving at the epithelial layer that experiences no collisions (Figs 2B and 3; S1 File).

### Estimating the hydrodynamic radius of an *n*-complex

The hydrodynamic radius of an *n*-complex is utilized directly in the calculation of the Smoluchowski encounter rate and indirectly in the calculation of the diffusion coefficient, which we calculate from the Stokes-Einstein relation. Since the Smoluchowski encounter rate is valid for spherical particle encounters, the equation may have some inaccuracy for complexes that are far from spherical. In particular, a linear configuration of particles gives a larger surface area relative to volume, increasing the probability of encounter. On the other hand, a more compact structure reduces viscous drag, increasing the diffusion coefficient and thus the encounter rate. Since the goal is to establish an upper bound on sIgA crosslinking, we utilize the larger hydrodynamic radius *r*_*n*_ = *nr*_1_ (where *r*_1_ is the radius of a single virion) for the hydrodynamic radius in the Smoluchowski relation while for the diffusion coefficient, we utilize the smaller hydrodynamic radius $\bar{r_{n}}=r_{1}\sqrt[{3}]{n}$ given by a spherical volume estimate. By the Stokes-Einstein relation, $D=\frac{k_{B}T}{6\pi \eta \bar{r_{n}}}$, where *k*_*B*_ is Boltzmann's constant, *T* is temperature, and *η* is the viscosity.

### Estimating the number of virions that experience no collisions

**Fig 4:**
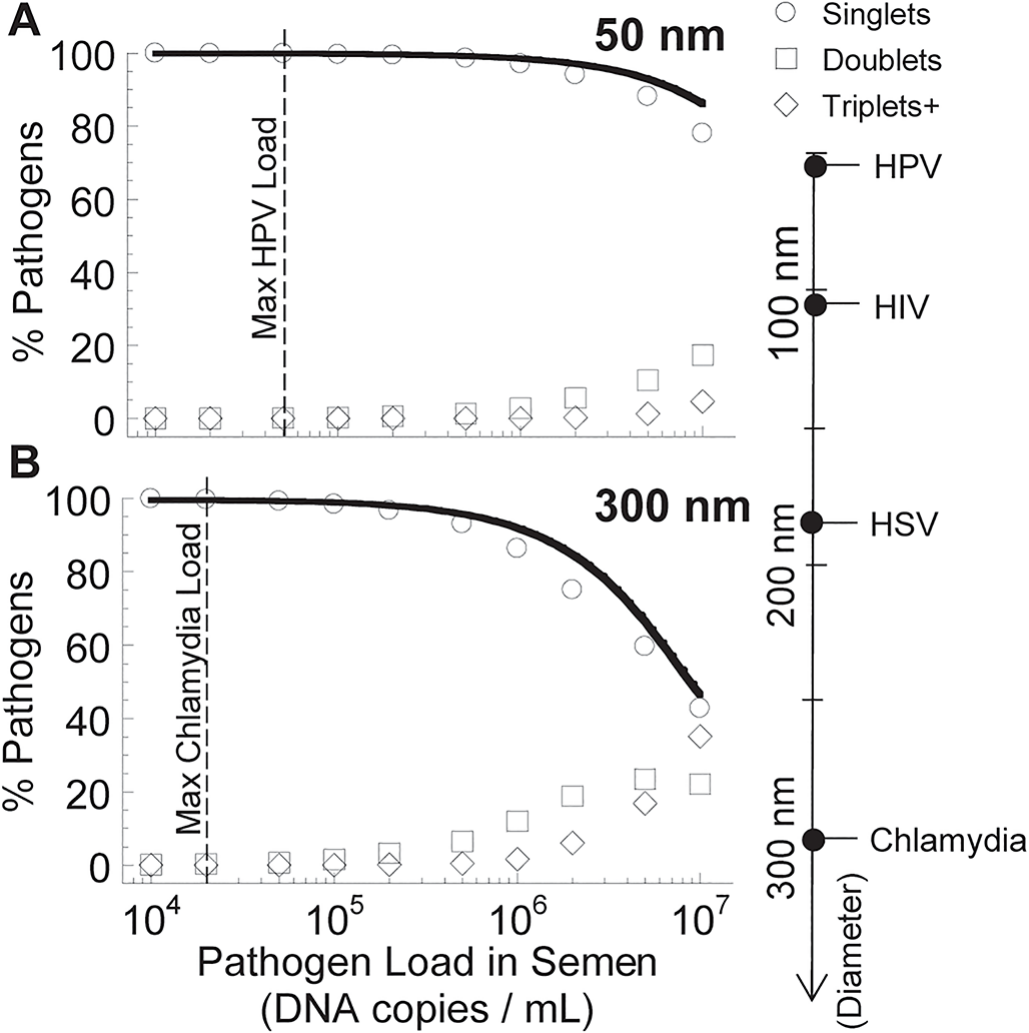
Estimating agglutination for pathogens of different size. Fraction of pathogens, (A) 50 nm and (B) 300 nm in diameter, that are predicted to diffuse from semen through CVM and reach the vaginal epithelium over the first 12 hours post semen deposition and which have undergone no collision (singlets), one collision (doublets) or two or more collisions (triplets+). The diffusivity of HPV in CVM is equivalent to that in buffer [17]; the diffusivity of Chlamydia, an obligate intracellular bacteria that lacks any active motility mechanism, is assumed to be the same in CVM as in buffer. The median and max HPV load in semen is 3.5 and 4.5 log_10_ copies per mL [18], whereas the median and max Chlamydia load in semen is 3.5 and 4.2 log_10_ copies per mL [19]. To enable rapid estimation of the viral load that may result in significant rates of agglutination, we also derived a mathematical equation (dashed curve; first summand of Eq 10) and utilized an exponential approximation (solid curve; Eq 3) that directly estimate the virion load arriving at the epithelial layer that experiences no collisions with other virions (see Appendix and S1 File).

In Figs 3 and 4 we display an analytical approximation for the percentage of virions that experience no collisions. This formula approximates the simulations from the reaction-diffusion system, and can be used to quickly estimate the extent of agglutination possible at known viral concentration. The key to developing an explicit formula in this estimate is to replace the real first passage time distribution with an exponential distribution with the analogous mean parameter. The rationale for this approximation is that the virions that are most likely to experience collisions are the ones that take the longest time to exit the region. While the exponential distribution does a poor job of approximating the passage time for the earliest exits, the match is excellent for the latest exits. Deviations occur at high concentrations though, slightly underestimating agglutination levels.

To express the result, we view the dynamics from the point of view of a single virion and compute the probability that it will not collide with any virions before passing through a layer. In the Appendix, we will justify the following approximation:

$$P\left\{\text{Particle experiences no collision},\;\text{given an exit before time}\;T\right\}\approx \frac{1-e^{-k_{S}V_{0}\bar{\tau }\left(1-e^{-T/\bar{\tau }}\right)}}{k_{S}V_{0}\bar{\tau }\left(1-e^{-T/\bar{\tau }}\right)},$$ (2)

where *k*_*S*_ is the Smoluchowski encounter rate, *V*_0_ is the initial viral concentration, *T* is the biological or experimental cutoff time, and

$$\bar{\tau }=\frac{1}{6D}\left(2L^{2}+2Ld-d^{2}\right)$$ (3)

is the population average passage time. That is, $\bar{\tau }$ is the expected amount of time for a virion with diffusivity *D* to pass through an interval [0, *L*] with absorbing boundary conditions at *z* = 0, reflecting boundary conditions at *z* = *L*, and initial distribution uniform over the semen layer [*d*, *L*].

## Results

### HIV virions rarely collide with each other at viral loads commonly found in genital secretions

For sIgA to protect by agglutinating a virus population, viruses must first encounter and collide with other virions before reaching target cells. Men who are acutely infected with HIV are most likely to transmit the infection due to elevated viral load in semen (i.e. 4.5 log_10_ RNA copies per mL [16]) compared to those of chronically infected men. Thus, we are particularly interested in evaluating the potential extent of sIgA-induced agglutination against HIV in semen of acutely infected individuals. Even before extensive modeling, we quickly suspected that HIV agglutination may be a rare event. According to the classical Smoluchowski encounter rate (see Materials and Methods for a full discussion), a 100 nm particle in water at the same 4.5 log_10_ concentration will encounter another particle at a rate of once per 500 hours. In comparison, the mean passage time by Brownian diffusion for such a particle through a 50 *μ*m thick mucus layer is a little over 3 mins. Similarly, Chandrasekhar found that collision rate, which naturally depends on the average spacing between virions, is proportional to the reciprocal of the cube root of the concentration [20]. A simple calculation yields an average spacing of 300 *μ*m, which is six times the thickness of the mucus layer; any collisions acting as a precursor to agglutination should therefore be exceedingly rare.

Nevertheless, these simple estimates do not provide insight into the extent of collision frequency and potential extent of agglutination on a *per virion* basis, which is necessary for us to begin to evaluate the extent of protection. We thus sought to develop a more complex model that better describes the dynamics of individual HIV virions during male-to-female vaginal transmission. We and others have previously described a mathematical model for characterizing the HIV diffusion across mucus secretions in the female reproductive tract [21–24]. From the moment semen is deposited in the vaginal lumen (time *T* = 0), the model tracks the diffusion of individual HIV virions from the semen layer (*d* < *z* < *L*) into the CVM layer (0 < *z* < *d*) and finally to the epithelium (*z* = 0) (Fig 1). We use reported rates of HIV diffusion in genital secretions as well as estimated thicknesses of the mucus and semen layers (Table 1, Fig 1). Using this model, we can tally the number of collisions for each virion over time as virions at a known initial concentration in semen diffuse into the mucus layer.

**Table 1:** Parameters and values incorporated into modeling HIV diffusion across the vaginal mucosa.

Parameter	Value	Reference(s)
**HIV-1**		
Diameter	100 nm	[25]
Diffusivity in semen and CVM	1.27 *μ*m^2^/s ^a^	[26]
Mean viral load in semen	4.5 log_10_ RNA copies per mL	[16]
Max viral load in semen	6.7 log_10_ RNA copies per mL	[16]
**Vagina**		
Surface area of lumen	145 cm^2^ ^b^	[27, 28]
Volume of luminal CVM	750 *μ*L	[29, 30]
Volume of semen	3.0 mL	[31]
Thickness of CVM layer	50 *μ*m ^c^	
Thickness of semen layer	200 *μ*m ^c^	

^a^ Geometrically averaged D_eff_ for HIV was previously measured to be 0.25 *μ*m^2^/s, but with substantial fraction of viruses exhibiting more rapid mobility. For the current analysis, we used 1.27 *μ*m^2^/s, which represented the top 25^th^ percentile of virus mobility; this is in reasonable agreement with a more recent study of HIV diffusion in genital secretions [13].

^b^ The mean surface area of the vagina in the native state was previously estimated to be ~90 cm^2^ by injection of vinyl polysiloxane casts vaginally. Alternatively, surface area of vaginal lumen may also be inferred by the surface area of erect penis (average ~200 cm^2^) assuming complete insertion into the vagina. We took the average from the two approaches.

^c^ Thickness of CVM is estimated by (Volume of CVM/Surface area of lumen); Thickness of semen layer is estimated by (Volume of semen/Surface area of lumen).

In order to establish an upper bound on the rate of agglutination, i.e., *biasing the model in favor of agglutination*, we make the *extreme* assumption that every collision event results in immediate agglutination. Even then, we find that the vast majority (99.8%) of viruses deposited into the vaginal lumen will *not* have collided even once with another virion over the course of the first 12 hours post sperm deposition (Fig 2A). Of the remaining virions that will have collided with another virion, virtually all of them will have collided with only one other virion. Consequently, all but 0.2% HIV virions capable of penetrating across the mucus layer are expected to do so as individual virions (Fig 2B). The remaining few virions will only have collided with one or two other virions, which will only increase their hydrodynamic radius by a factor of 2 and thereby knockdown their diffusion constant by the same factor.

The frequency of collision is naturally dependent on the viral load in semen: the more HIV virions present, the more likely there will be virion encounters. To explore the significance of viral load on agglutination, we simulate a range of viral loads in semen to identify a threshold viral concentration where collisions between HIV virions become significant. Even at the upper range of HIV viral load in acutely infected individuals (i.e. 6.67 log_10_ RNA copies per mL), our model reveals that less than 20% of all HIV viruses will have collided with another HIV virus after 12 hours (Fig 3A). Furthermore, because the highest concentration of HIV virions is found in semen at the moment of vaginal deposition, the probability that a virion will encounter another virion is substantially reduced as that particular virion diffuses into the CVM layer (i.e. away from semen). Hence, the virions that most quickly penetrate into and diffuse across the mucus layer are also least likely to be agglutinated. This analysis explains why even at the highest HIV load, less than 17% of the virions that penetrate CVM within the first 12 hours post-deposition will have collided with at least one other virion (Fig 3B). In the majority of vaginal HIV transmission events, the extent of viral collision is most likely much less.

### Further reasons why HIV in mucus are unlikely to form large HIV/sIgA complexes

Despite the modest increase observed in the collision count and fraction of HIV virions that have collided with another virion at the exceedingly high viral concentrations, the true agglutinated fraction must be substantially if not orders of magnitude lower. First, a successful bond formation between an antibody and an antigen requires on the order of 10^3^ collisions on average [32]. Second, the Env trimers that most HIV binding antibodies target are only present with sparse density on the virus surface [25], which means the majority of the HIV surface will not agglutinate upon collision. Third, for sIgA to crosslink two HIV virions, sIgA must either be already bound to one virion *and* encounter a sIgA-free Env on a colliding virion, or simultaneously bind two sIgA-free Env on two colliding virions. Clearly, only a subset of virions will experience either. Altogether, this suggests that even if it is possible to deliver or induce anti-HIV sIgA in mucus coating the female reproductive tract with exceedingly high affinity to HIV-Env, sIgA-induced agglutination is unlikely to be a dominant mechanism of protection against male to female HIV transmission in humans.

### Minimal sIgA-induced agglutination for other sexually transmitted viruses?

The surprisingly low agglutination rate for HIV-sized viruses led us to evaluate whether poor agglutination is a unique feature of HIV (potentially as an evolutionary trait to evade mucosal immunity), or if it is also characteristic of other sexually transmitted viral infections. To do so, we simulate the collision rate of different sized spheres in the range of 50–300 nm over different concentrations; this size range encompasses virions such as 55 nm Human Papillomavirus (HPV), 180 nm Herpes Simplex Virus (HSV), and the intraceullar-obligate but non-mobile bacteria Chlamydia (300 nm) (Table 2). We found that the number of collisions increased slightly with particle radius but the collision rate remained minimal across the physiologically relevant range of viral load (Fig 4). This suggests significant agglutination may likely be prominent only at much higher concentrations more commonly associated with bacterial pathogens, and/or with pathogens that possess alternative mechanisms of motion (e.g. flagella-driven motility, chemotaxis), resulting in increased frequency of encountering another pathogen.

**Table 2:** Parameters and values for other sexually transmitted pathogens.

Parameter	Value	Reference(s)
**HPV**		
Diameter	55 nm	[18]
Diffusivity in semen and CVM	11.9 *μ*m^2^/s ^a^	[17]
Mean load in semen	3.5 log_10_ copies per mL	[18]
Max load in semen	4.5 log_10_ copies per mL	[18]
**Chlamydia**		
Diameter	300 nm	[19]
Diffusivity in semen and CVM	2.17 *μ*m^2^/s ^a^	Calculated from Stokes-Einstein law
Median load in semen	3.5 log_10_ copies per mL	[19]
Max load in semen	4.2 log_10_ copies per mL	[19]

^a^ The diffusivity was calculated from the Stokes-Einstein law in water, correct for HPV [17]. Diffusivity has not been measured for chlamydia, thus we assumed its diffusivity in water.

## Discussion

The low agglutination frequency predicted may appear to contradict earlier *in vivo* studies that showed sIgA confers improved protection compared to IgG and IgA against viral infections, as well as previous studies that illustrate sIgA-induced agglutination of sperm. For example, topically applied dimeric IgA resulted in enhanced protection against rectal R5 SHIV transmission compared to IgG_1_ with comparable neutralizing activity *in vitro* [33]. One possible difference may be attributed to the relatively high dose of SHIV used in many rectal and vaginal challenge studies, which would substantially increase the likelihood of agglutination. Even in studies that measure protection against repeated low-dose challenges, the infectious titers used are still substantially greater than physiologically relevant titers in human vaginal transmission. While sIgA can readily agglutinate sperm, it should be noted that sperm is markedly different than HIV in concentration (~10^7^–10^8^ sperm per mL vs. ~10^4^–10^6^ HIV per mL), dimension (the head of sperm is ~3x5 *μm* vs. ~100 nm for HIV), and mechanism of motility (active motion vs. passive Brownian diffusion). Since each of these factors increases the collision frequency for sperm relative to HIV in semen, we believe sIgA-induced agglutination of sperm should not be used as a basis for assuming that sIgA can also agglutinate HIV under physiologically-relevant conditions. It is also important to point out that sIgA may protect by other mechanisms than agglutination. For example, a greater inter-Fab distance for dimeric IgA may enable more efficient bivalent binding (i.e., greater avidity) of a larger fraction of the sparse Env trimers on HIV-1 virions than IgG. The higher degree of glycosylation in sIgA may also trigger other mechanisms of innate immunity [34]. While we found that endogenous, HSV-specific IgG in CVM of women with largely *Lactobacilli*-dominated microflora were sufficiently stable to readily bind and immobilize HSV virions [12], improved antibody stability may be relevant in CVM from women with vaginal microbiota that produce substantial quantities of proteases and glycosidases, such as microbes associated with bacterial vaginosis [35]. Last but not least, sIgA may effectively agglutinate HIV-1-infected lymphocytes and help protect against mucosal transmission by cell-associated HIV viruses.

An emerging mechanism of mucosal immune protection is the immobilization of individual viruses due to antibody-mucin interactions, which in turn prevent viral translocation across mucus. The interactions between antibodies and mucus are generally low-affinity and transient; for example, the diffusion of IgG and IgA molecules (diameter 10 nm) in human mucus (pores 340 ± 50 nm [36]) is slowed only 5%-20% compared to buffer [17, 37]. We have recently shown that this seemingly negligible affinity is sufficient for both endogenous and exogenously added HSV-specific IgG to trap HSV-1 virions in human CVM with sub-neutralizing potency, presumably because the array of virion-bound IgG ensures a sufficient number of transient low-affinity bonds between the virus and mucins at any given time [12]. Since IgA molecules possess similar mucin affinity to IgG [17, 37], virions may very well be immobilized by a collection of surface-bound sIgA. sIgA could confer an added advantage to mucosal protection in CVM compared with IgG if the O-glycans-rich SC can interact more strongly with mucins than the low-affinity observed between individual IgG/IgA and the predominantly Muc5B mucins found in CVM. For example, Phalipon *et al*. previously suggested that SC may associate with mucus lining and enhanced IgA-mediated protection against *S*. *flexneri* in the lung (note: lung mucus consists of primarily Muc5B mucins, similar to genital secretions) [38]. Nevertheless, it is not obvious whether a stronger bond between individual antibody molecules and mucins would necessarily enhance trapping potency, since an antibody that is stably anchored to mucins would possess greatly limited range of motion that consequently reduces its binding rate to an antigen target. Unfortunately, much remains unknown about the process by which sIgA can dynamically block microbial invasion across a mucus layer [3]. Given the complexity and number of confounding contributing factors, a systemic experimental study comparing the ability of IgG and sIgA to block virus and bacteria translocation across mucus is needed, and would no doubt clarify the potential role of sIgA-induced agglutination in vaginal immune protection.

The long held notion that sIgA is critical to mucosal protection is based in part on the massive amount of sIgA secreted along the GI tract relative to IgG. In contrast, we generally measure a ratio approaching 1:1 to 1:2 for IgG:sIgA in respiratory secretions in our lab (unpublished observations) and a ratio in excess of 10:1 or more (in favor of IgG) in genital tract secretions. The markedly higher sIgA levels in the gut suggests sIgA may be adapted to offer enhanced protection at the intestinal mucosa relative to other mucosal surfaces. A possible explanation may be attributed to the immense number of motile microbes in the gut, which most likely results in markedly higher collision frequency–a first step in sIgA-induced agglutination. Likewise, many bacteria share homology with respect to their surface proteins (e.g., lipopolysaccharides between *Salmonella typhimurium* vs. *Escherichia coli*). This suggests that the same sIgA molecule may bind to multiple different bacteria, leading to further increase in the rates and prevalence of agglutination [39]. Last but not least, sIgA-mediated agglutination can offer improved trapping potency compared to IgG. IgG-mediated trapping of individual highly motile microbes is likely to require many dozens if not hundreds of pathogen-bound IgG. In contrast, as few as one single sIgA can agglutinate two bacteria, and the minimum number of sIgA required to agglutinate multiple pathogens is likely to scale directly with the order of pathogen number (e.g. two sIgA molecules can agglutinate three bacteria). Since agglutination markedly suppresses the mobility of a bacterium, we speculate that sIgA likely offers an exceptionally efficient mechanism of protection against dense populations of intestinal bacterial microbes. sIgA has also been implicated in the protection against gastrointestinal viruses such as poliovirus, norovirus and rotavirus. It remains to be determined experimentally whether this is due to the highly efficient process for local sIgA production and secretion in the GI tract relative to IgG, markedly higher viral titer for these viruses compared to sexually transmitted viruses, or if sIgA can confer additional protection by other mechanisms.

## Appendix

We begin our derivation of the approximate formula (2) by recording probability that a particle experiences no collisions as of time *t* with a fixed particle concentration (i.e., ignoring that concentration changes due to particles exiting the domain). Let *N*(*t*) denote the number of collisions that have occurred as of time *t*. Then, modeling collisions as a Poisson arrival process with a rate being the product of the Smoluchowski encounter rate and the fixed viral concentration, *k*_*S*_ *V*_0_, we have that

$$P\left\{N\;\left(t\right)=0\right\}=\text{exp}\left(-k_{S}V_{0}t\right)$$ (4)

For a population of particles with high diffusivity, however, there can be a significant alteration in concentration over time as the particles leave the system. This leads to differing collision rates and significantly different collision counts for particles in the system in comparison with those that have exited (i.e., particles that have left the system do not subsequently collide). Thus, we examine the case with a particle concentration that changes over time. To this end, assume that all virions have diffusivity *D* and radius *r*, while the semen-CVM system has width *L*. Define *τ* to be the random time it takes for the virion to pass through the layer. Then the first passage time density is given by

$$\rho \left(t\right)=\frac{2D}{L(L-d)}\sum_{k=0}^{\infty }\text{cos}\frac{\left(2k+1\right)\pi d}{2L}\text{exp}\left[-\frac{{\left(2k+1\right)}^{2}\;\pi^{2}D}{4L^{2}}t\right],$$ (5)

where *ρ*(*t*) can be derived by solving the 1D heat equation on the interval [0, *L*] with the given initial and boundary conditions [40]. The *survival function*, F(t): = **P**{*τ* > *t*}, can be expressed in terms of the passage time density,

$$F\left(t\right)=1-\int_{0}^{t}\rho \left(t'\right)\;dt'.$$ (6)

In this setting, we model the collisions by Poisson arrival process with a time-dependent intensity given by the Smoluchowski encounter rate, *λ*(*t*) = *k*_*S*_ *V*_0_ *F* (*t*), where we recall that *k*_*S*_ = 16*πDr*, *V*_0_ is the initial viral concentration, and *F* (*t*) is the fraction of virions remaining in the solution at time *t*. It follows that

$$P\left\{N\left(t\right)=0\right\}=\text{exp}\left(-\int_{0}^{t}k_{S}V_{0}F\left(t\prime \right)\;dt\prime \right)$$ (7)

Using the law of total probability, we have an exact formula for the probability that a given virion does not encounter any others before exiting:

$$\begin{array}{l} P\left\{N\left(\tau \right)=0\right\}=\int_{0}^{\infty }P\left\{N\left(\tau \right)=0\;|\tau =t\right\}\rho \left(t\right)\;dt\\ \;=\int_{0}^{\infty }\text{exp}\left(-\int_{0}^{t}k_{S}V_{0}F\left(t\prime \right)\;dt\prime \right)\rho \left(t\right)\;dt. \end{array}$$ (8)

If there is an external cutoff time *T* (imposed by drainage of CVM, for example), we modify the above formula as follows.

$$\begin{array}{l} \;P\left\{N\left(\text{min}\left(T,\tau \right)\right)=0\right\}=P\left\{N\left(\tau \right)=0,\tau <T\right\}+P\left\{N\left(T\right)=0,\tau \geq T\right\}\\ =\int_{0}^{T}P\left\{N\left(\tau \right)=0\;|\tau =t\right\}\rho \left(t\right)dt+P\left\{N\;\left(T\right)=0\right\}\int_{T}^{\infty }\rho \left(t\right)dt\\ \;=\int_{0}^{T}\text{exp}\left(-\int_{0}^{t}k_{S}V_{0}F\left(t\prime \right)dt'\right)\rho \left(t\right)dt+\text{exp}\left(-\int_{0}^{T}k_{S}V_{0}F\left(t\prime \right)dt\prime \right)\int_{T}^{\infty }\rho \left(t\right)dt. \end{array}$$ (9)

### Estimate of first-passage times

In order to derive a closed-form estimate for Eq (10), we approximate the passage time distribution with an exponential distribution of the same mean, calculated below. Indeed, this approximation is justified in that the viral population entering the epithelial layer closely matches the exponential approximation (Figs 3 and 4). Using tildes to denote the analog density $\tilde{\rho }$ and survival function $\tilde{F}$, we have

$$\tilde{\rho }\left(t\right)=\frac{1}{\mu }e^{-t/\mu }\;\text{and}\;\tilde{F}\left(t\right)=e^{-t/\mu }.$$ (10)

The modified version of Eq (9) can be evaluated to give

$$P\left\{N\left(\tau \right)=0\right\}\approx \frac{1}{k_{S}V_{0}\mu }\left(1-e^{-k_{S}V_{0}\mu }\right).$$ (11)

With an external cutoff time T, the modified first summand of Eq (10) is given by

$$P\left\{N\left(\tau \right)=0,\tau <T\right\}\approx \frac{1}{k_{S}V_{0}\mu }\left(1-e^{-k_{S}V_{0}\mu \left(1-e^{-\frac{T}{\mu }}\right)}\right).$$ (12)

Meanwhile, the second summand is approximated as

$$\begin{array}{c} P\left\{N\left(T\right)=0,\tau \geq T\right\}\approx e^{-\int_{0}^{T}k_{S}V_{0}\tilde{F}\left(t\prime \right)dt'}\int_{T}^{\infty }\tilde{\rho }\left(t\right)\;dt\\ =e^{-k_{S}V_{0}\mu \left(1-e^{-T/\mu }\right)}e^{-T/\mu }. \end{array}$$ (13)

Together,

$$P\left\{N\;\left(\text{min}\;\left(T,\tau \right)\right)=0\right\}\approx \frac{1}{k_{S}V_{0}\mu }-\left(\frac{1}{k_{S}V_{0}\mu }-e^{-\frac{T}{\mu }}\right)e^{-k_{S}V_{0}\mu \left(1-e^{-\frac{T}{\mu }}\right)}.$$ (14)

Conditioning on the population of virions that have entered the epithelial layer, Eq (3) is obtained with the mean passage time μ (for a virion at a fixed initial location) in place of $\bar{\tau }$, the population average passage time.

$$P\left\{N\;\left(T\right)=0\;|\tau <T\right\}=\frac{P\left\{N\;\left(T\right)=0,\tau <T\right\}}{P\left\{\tau <T\right\}}=\frac{1-e^{-k_{S}V_{0}\mu \left(1-e^{-T/\mu }\right)}}{k_{S}V_{0}\mu \left(1-e^{-T/\mu }\right)}.$$

### Calculating the population average passage time

In order to calculate $\bar{\tau }$, the population average passage time, we consider particles diffusing through the interval [0, *L*] where 0 is absorbing and *L* is reflecting. The initial condition is distributed over a subinterval [*d*, *L*] and we wish to compute the probability of particles diffusing through the layer with no encounters. A first-order approximation is given by computing the expected exit time from the interval [0, 2*L*] with initial condition uniformly distributed over [*d*, 2*L* − *d*]. The expected time to exit [0, 2*L*] from the initial position *x*, denoted *u*(*x*), satisfies the ODE

Du″(x)=−1,u(0)=u(2L)=0, (15)

which has solution $u\left(x\right)=\frac{1}{2D}x\left(2L-x\right)$. Now, integrating over the region of the initial condition, we obtain Eq (4):

$$\bar{\tau }=\frac{1}{6D}\left(2L^{2}+2Ld-d^{2}\right).$$

## Supporting Information

S1 File Analytical estimate of virions arriving at epithelial layer without collisions. The Excel file gives an estimate of the virions arriving at the epithelial layer having experienced no collisions (Eq 3), based on user-given input parameters.
(XLSX)
